# 

**Published:** 1839-12-28

**Authors:** 


### THE MEDICAL EXAMINER.

PHILADELPHIA, DECEMBER 28, 1839.

### WORKS RECEIVED.

DR. GROSS'S PATHOLOGICAL ANATOMY.—We have received a copy of this excellent work, and shall review it in one of the earlier numbers of the ensuing year. From the examination which we have given to the work, we can recommend it to the profession, as the most complete treatise on pathological anatomy within their reach. It possesses, indeed, some advantages not to be met with in other works on the subject.

INTRODUCTORY LECTURES, by Professors Hun, of Albany; Mitchell, of Transylvania; and Pancoast, of Philadelphia—Suitable, well written addresses, and entirely adapted to the purposes of introductory lectures.

THE NURSE'S GUIDE, "*a series of instructions to females who wish to engage in the important business of nursing mother and child in the lying-in chamber.* By J. WARRINGTON, M.D., Lecturer on Practical Obstetrics. Philadelphia: Thomas, Cowperthwait & Co. 1839.

Dr. Warrington is engaged in the practice and practical teaching of Obstetrics, and is therefore conversant with the defective knowledge of many of the women who undertake the responsible office of nurse to lying-in women. His instructions are practical, and written in the plain, intelligible style which is adapted for the purposes of the work.

Documents on the Yellow Fever.—We have received several valuable memoirs on this subject. 1st, a manuscript memoir from Dr. Rufz, of Martinique, for the Medical Examiner; this is an elaborate and very complete history of the disease as it appeared in that island. It will be published either entire, or in the form of copious extracts, in the early numbers of the Examiner.

We have also received two printed documents, one from the physicians of Augusta, and another from Dr. Ashbel Smith of Texas, detailing the symptoms and peculiarities of the disease as it occurred in these situations. An abstract of their contents will appear as soon as practicable.

